# Impact of Albumin Leakage on the Mortality of Patients Receiving Hemodialysis or Online Hemodiafiltration

**DOI:** 10.3390/jcm13071865

**Published:** 2024-03-24

**Authors:** Manabu Tashiro, Kazuyoshi Okada, Yusaku Tanaka, Hiroyuki Michiwaki, Hisato Shima, Tomoko Inoue, Toshio Doi, Jun Minakuchi

**Affiliations:** 1 Department of Nephrology, Kawashima Hospital, 6-1 Kitasakoichiban-Cho, Tokushima-City 770-0011, Japan; kokada@minos.ocn.ne.jp (K.O.); h.shima@khg.or.jp (H.S.); t.inoue@khg.or.jp (T.I.); t.doi@khg.or.jp (T.D.); minakuchi_j@me.com (J.M.); 2 Department of Clinical Engineering, Kawashima Hospital, 6-1 Kitasakoiciban-Cho, Tokushima-City 770-0011, Japan; yu-tanaka0724@khg.or.jp (Y.T.); michiwaki@khg.or.jo (H.M.)

**Keywords:** hemodialysis, online hemodiafiltration, albumin leakage, mortality, solute removal

## Abstract

**Background**: Online hemodiafiltration (OHDF) has a lower mortality rate than hemodialysis (HD). We aimed to investigate the impact of the albumin leakage on the mortality of patients receiving HD or OHDF. **Methods**: In this single-center study, consecutive patients receiving renal replacement therapy between January and April 2018 were retrospectively registered. Using (1:1) propensity score matching, 3-year all-cause mortality was compared between patients receiving HD and OHDF, and the impact of albumin leakage on the mortality rate in both groups was investigated. **Results**: Of the 460 patients, 137 patients receiving HD were matched with an equal number of patients receiving OHDF. OHDF was associated with higher albumin leakage (*p* < 0.001) and a lower mortality than HD (log-rank test, *p* < 0.001). Albumin leakage was associated with mortality in patients receiving HD (per 1 g increase, hazard ratio (HR): 0.495, 95% confidence interval (CI): 0.275–0.888) and patients receiving OHDF (per 1 g increase, HR: 0.734, 95% CI: 0.588–0.915). Patients receiving HD, with the highest albumin leakage tertile (>3 g), had a similar mortality rate to patients receiving OHDF, with similar albumin leakage. **Conclusions**: The negative relationship between albumin leakage and mortality suggests the benefit of removing middle- to -large-molecular-weight substances to improve survival.

## 1. Background

The number of patients receiving OHDF is increasing worldwide [1]. Almost half of patients receiving hemodialysis are now receiving HDF in Japan; there are now more than 120,000 patients treated with online hemodiafiltration, over 90% of whom use pre-dilution online hemodiafiltration (pre-OHDF).

In Japan, pre-OHDF is usually used because of its lower blood flow rate, requiring a rate of 200–300 mL/min, whereas post-OHDF requires more than 300 mL/min.

A high blood flow rate is unsuitable for Japanese people because of their small physique, which is why pre-OHDF is preferred.

The JRDR has compared the 1-year prognosis between patients receiving pre-OHDF and those receiving HD by using propensity score matching of the data in the national database. High-volume pre-OHDF has been associated with a reduced mortality risk and a reduced cardiovascular risk [2,3], as well as better long-term prognosis with substitution volumes of more than 40 L [2].

There have been various reports worldwide on the usefulness of OHDF. The ESHOL study showed a reduction in all-cause mortality with HDF [4].

However, in a Turkish study [5] and the Dutch CONTRAST [6], it was only useful with the high convection volume of HDF. Therefore, the mechanisms underlying HDF that may improve the long-term prognosis compared to HD are not clear.

OHDF can mainly remove a wide range of middle- to large-molecular-weight solutes that cannot be adequately removed by HD. The enhanced removal of these uremic toxins may be associated with the relief of symptoms and improved quality of life.

High-volume OHDF further increases the removal efficiency.

The removal of medium-sized solutes with a molecular weight above 17 kDa is associated with albumin leakage [7,8]. Therefore, dialysis conditions with albumin leakage imply the removal of middle- to large-molecular-weight uremic substances. In recent years, uremic toxins with larger molecular weights have been targeted [9,10], but the evidence is still lacking. In addition, there are no indicators of the removal efficiency for middle- to large-molecular-weight substances.

We hypothesized that the active removal of middle- to large-molecular-weight substances would be associated with a better prognosis. Therefore, we aimed to investigate the association between albumin leakage and prognosis in patients receiving HD and OHDF.

## 2. Methods

### 2.1. Retrospective Observational Study

#### 2.1.1. Patient Selection

We selected individuals from the 521 patients undergoing maintenance dialysis with HD or OHDF at Kawashima Hospital as of 1 April 2018. Patients with substitution volumes of 40 L (*n* = 6) and 100 L (*n* = 1) in pre-OHDF and 6 L (*n* = 1), 14 L (*n* = 1) and 15 L (*n* = 1) in post-OHDF, intermittent infusion hemodiafiltration patients (i-HDF, *n* = 37), and patients in the hospital or who did not provide consent (*n* = 14) were excluded.

Patients with i-HDF were excluded because the albumin leakage could not be assessed, and the substitution volume group with a small number of patients was also excluded. This study analyzed only one facility of the Kawashima Hospital Clinic, with 460 patients, including 159 who were receiving HD and 301 who were receiving OHDF. The baseline characteristics, such as age, sex, dialysis conditions, dialysis efficiency (Kt/V), presence of diabetes, body mass index (BMI), pre-dialysis systolic blood pressure (sBP), regular pre- and post-dialysis blood samples, and normal protein catabolic rate (nPCR) were obtained from medical records.

#### 2.1.2. Dialysis Conditions

The dialysis settings in our hospital were as follows: total dialysate flow rate (QD): 500 mL/min, quantity of blood flow (QB): 250–300 mL/min, with substitution volumes of 60 L, 72 L, and 84 L for pre-OHDF and 8 L, 10 L, 12 L, and 16 L for post-OHDF, membrane area: 1.5–3.0 m^2^, and dialysis time: 4–5 h.

The dialysis membranes used are listed in Table 1.

This research was conducted under only one point of blood sampling and dialysis conditions and did not consider later changes to the dialysis conditions.

### 2.2. Albumin Leakage

Albumin leakage was measured by collecting the total dialysis effluent from each dialyzer or hemodiafilter for 4 h, which was averaged according to the substitution volume. The QB was 250 mL/min for HD and 280 mL/min for OHDF; the dialysate flow rate (QD) was 500 mL/min for HD and 500 mL/min for OHDF. The substitution volumes were 60, 72, and 84 L pre-OHDF and 8, 10, 12, and 16 L post-OHDF. The dialysis machine and hemodialysis filters used in this study and the mean albumin leakage rates are listed in Supplementary Table S1.

#### 2.2.1. Statistical Analysis

To analyze the effects of HD and OHDF on the survival outcomes, the propensity scores were matched in 137 pairs of patients receiving HD and OHDF.

Then, we compared the survival between the HD group and the OHDF group using propensity score matching.

**Figure 1:**
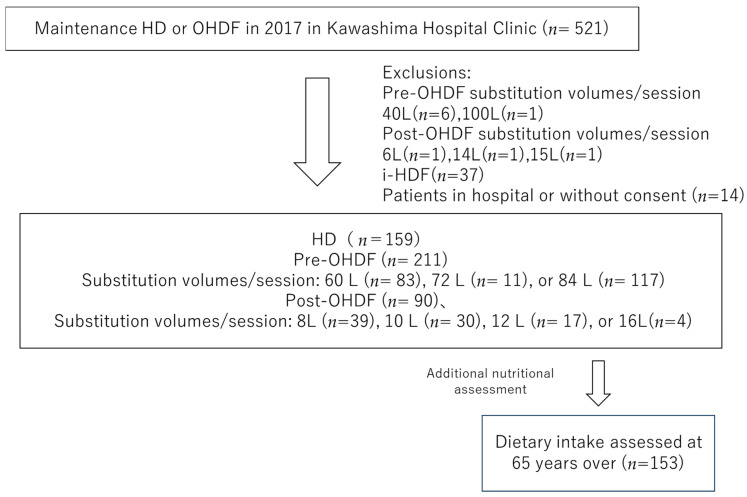
Patient selection.

The patients treated with OHDF were further divided into a pre-OHDF group (*n* = 211) and a post-OHDF group (*n* = 90) (Figure 1).

Then, we used the log-rank test to compare the Kaplan–Meier survival curves, which compared the effect of albumin leakage on 3-year all-cause mortality. In the pre-OHDF group, the association between the substitution volumes of 60 L (*n* = 83), 72 L (*n* = 11), and 84 L (*n* = 117) and long-term prognosis was also investigated.

The following 13 items were used to calculate the propensity score for comparing patient survival outcomes: age, dialysis vintage, presence or absence of diabetes mellitus, body mass index (BMI), systolic blood pressure (sBP), hemoglobin (Hb), albumin (Alb), Kt/V, calcium (Ca), phosphate (P), β2-microglobuline (β2-mg), normal protein catabolic rate (nPCR), and high-sensitivity C-reactive protein (Hs-CRP).

Survival analysis of the groups was performed using the Kaplan–Meier method. Statistical significance between the groups was determined with the log-rank test and Cox proportional hazard.

All analyses were performed using SPSS Statistics for Windows, version 26 (IBM, Armonk, NY, USA), and *p* < 0.05 was considered statistically significant.

#### 2.2.2. Subgroup Analysis

In addition, 153 hemodialysis patients over 65 years of age who were included in the current target group and whose dietary intake was assessed in an interview with a dietitian were also analyzed.

The reason for selecting patients over 65 years of age is because sarcopenia and nutritional disorders increase in these hemodialysis patients [11].

To assess dietary intake, the patients were asked to describe their diet for three days. From this information, a dietitian calculated the total calorie, protein, phosphorus, potassium, and salt intake.

We examined whether dietary intake was associated with albumin leakage, etc.

In addition, we looked at the prognostic impact of albumin leakage in the groups over 80 years of age and 60~79 years of age.

## 3. Results

### 3.1. Comparison of Patient Survival Outcomes between the HD and OHDF Groups

The HD group was an older and less well-nourished group of patients with a shorter vintage of dialysis, lower albumin leakage, and worse prognosis than the OHDF group.

The patients’ backgrounds before and after propensity score matching are shown in Table 1.

The long-term prognosis of 137 pairs of patients in the HD group and OHDF group obtained by propensity score matching was compared.

**Figure 2:**
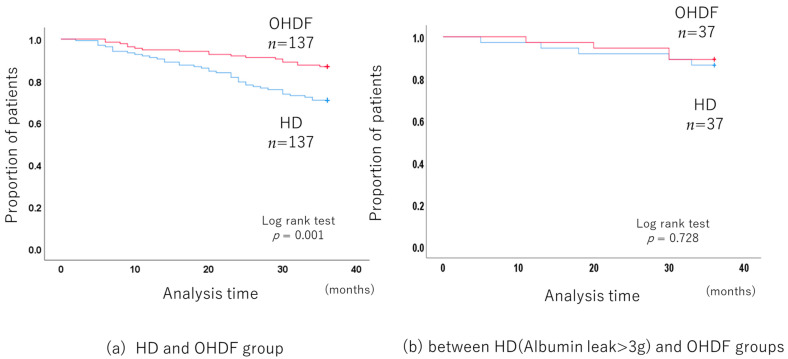
Comparison of the long-term prognosis between the HD and OHDF groups after propensity score matching.

The 3-year all-cause mortality was significantly lower in the OHDF group than in the HD group (*p* < 0.001, log-rank test) (Figure 2a), with a hazard ratio (HR) of 0.409 (95% confidence interval (CI): 0.234–0.713).

The albumin leakage was significantly higher in the OHDF group than in the HD group (means: 4.38 g/session vs. 2.23 g/session, *p* < 0.001) (Supplementary Table S2).

Propensity score matching, including albumin leakage, no longer showed a benefit for the OHDF group (*p* = 0.095).

In addition, patients receiving HD who had albumin leakage of more than 3 g (albumin leakage: 3.92 g/session ± 0.46) had a similar mortality rate to patients receiving OHDF and similar albumin leakage (albumin leakage: 4.01 g/session ± 1.55) (*p* = 0.713, log-rank test) (Figure 2b).

No dropouts were observed in the patients included in the current study.

### 3.2. Comparison in the HD Group

The mean albumin leakage was 2.14 g ± 1.16, with a median of 1.92 g.

The albumin leakage was stratified into three levels: less than 1 g, 1–3 g, and more than 3 g.

The patients’ characteristics related to the albumin leakage level are shown in Supplementary Table S3.

The group with higher albumin leakage had a better long-term prognosis because they were younger and better nourished.

**Figure 3:**
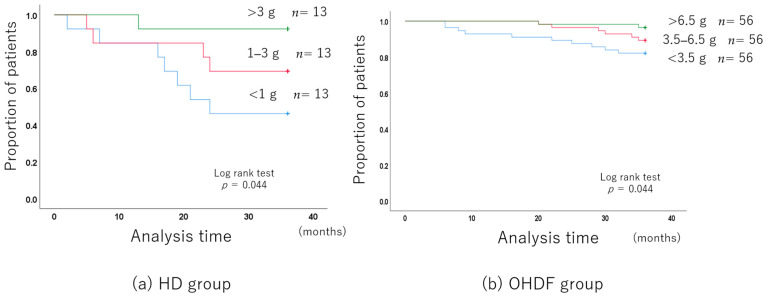
Long-term prognosis associated with the albumin leakage level.

The 3-year all-cause mortality improved with the increase in albumin leakage, even after adjusting for background factors (Figure 3a).

Albumin leakage was associated with mortality in patients receiving HD (per 1 g increase, hazard ratio (HR): 0.495, 95% confidence interval (CI): 0.275–0.888).

### 3.3. Comparison in the OHDF Group

#### 3.3.1. OHDF Group

The mean albumin leakage rate was 4.92 g ± 2.79, with a median of 4.4 g.

The albumin leakage rates were stratified into three levels: less than 3.5 g, 3.5–6.5 g, and more than 6.5 g.

The patients’ characteristics at each albumin leakage level are shown in Supplementary Table S4.

The long-term prognosis was better in the group with higher albumin leakage even before propensity score matching.

After adjusting for the background factors, the 3-year all-cause mortality was still significantly better with higher albumin leakage (Figure 3b).

Albumin leakage was associated with mortality in patients receiving OHDF (per 1 g increase, (HR): 0.734, 95% (CI): 0.588–0.915).

#### 3.3.2. Pre-OHDF Group

For the pre-OHDF group, the results were examined not only in terms of the albumin leakage but also in terms of the substitution volume.

The mean albumin leakage rate was 4.13 g ± 2.38, with a median of 3.4 g.

The albumin leakage rates were classified into three levels, as in the OHDF group: less than 3.5 g, 3.5–6.5 g, and more than 6.5 g.

The long-term prognosis was better in the group with higher albumin leakage, although the patient backgrounds differed.

**Figure 4:**
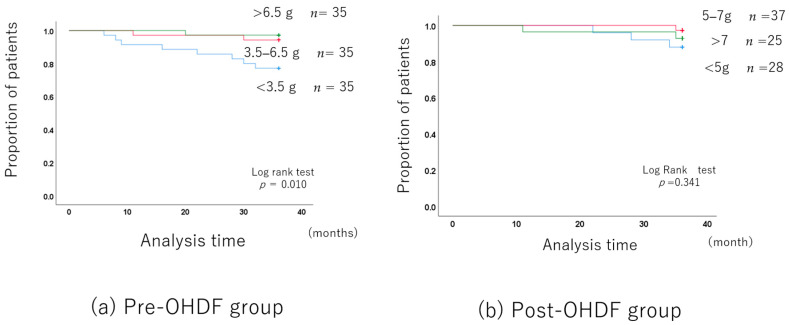
Albumin leakage and long-term prognosis in the OHDF group.

However, after adjusting for the patient backgrounds (Supplementary Table S5), there was no difference in the long-term prognosis between the 3.5–6.5 g albumin leakage group and the group with more than 6.5 g. Of course, the long-term prognosis was significantly better than in the group with less than 3.5 g (Figure 4a).

The substitution volume and prognosis were also examined, but there were no significant differences among the three groups of 60 L, 72 L, and 84 L (Supplementary Figure S1).

The only background factor with a significant difference was albumin leakage.

The mean albumin leakage rates were 3.47 ± 2.48, 5.85 ± 4.34, and 4.44 ± 1.88 for the 60 L, 72 L, and 84 L substitutions, respectively, whereas the median albumin leakage rate was 3.8 g for the 60 L, 3.5 g for the 72 L, and 3.4 g for the 84 L substitutions (Supplementary Table S6).

The ranges of albumin leakage was 0.8–11.1 g for the 60 L substitution volume, 2.8–14.1 g for the 72 L substitution volume, and 0.9–7.0 g the for 84 L substitution volume.

#### 3.3.3. Post-OHDF Group

The mean albumin leakage rate was 6.77 g ± 2.83, with a median of 5.9 g.

The albumin leakage rates were classified into three levels: less than 5 g, 5–7 g, and more than 7 g.

The albumin leakage was 4.04 ± 0.70 in the group with less than 5 g, 6.18 ± 0.47 in the 5–7 g group, and 9.96 ± 13.70 in the group with more than 7 g (Supplementary Table S7).

The long-term prognosis did not differ among the three albumin leakage groups (Figure 4b).

### 3.4. Subgroup Analysis in Patients Age > 65 Years

In total, 153 patients over 65 years of age who were able to assess their dietary intake were included.

In terms of the dietary intake, four items were significantly positively correlated each other.

**Figure 5:**
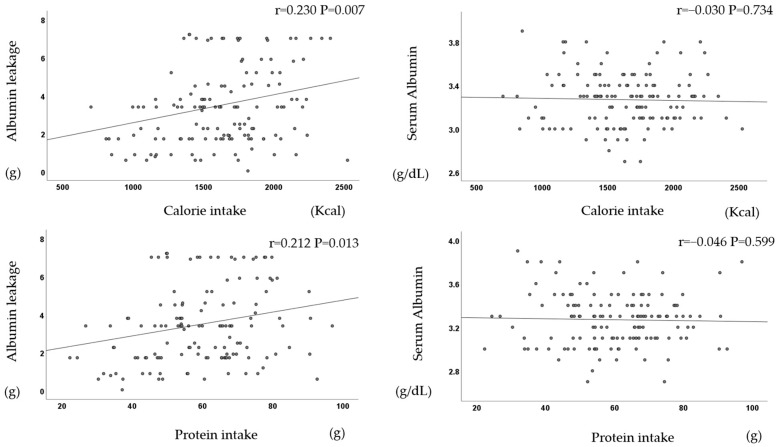
Dietary intake correlates with albumin leakage but not with serum albumin.

There was a significant positive correlation between dietary intake (calories, protein, phosphorus, and potassium) and albumin leakage but not serum albumin (Figure 5).

The 3-year prognosis was significantly better in patients with a higher dietary intake of calories, protein, phosphorus, and potassium.

In addition, the 3-year prognosis was positively correlated with albumin leakage (*p* = 0.003), calorie intake (*p* = 0.014), and BMI (*p* = 0.041), and it was negatively correlated with age (*p* <0.001), but it was not correlated with serum albumin (*p* = 0.257).

**Figure 6:**
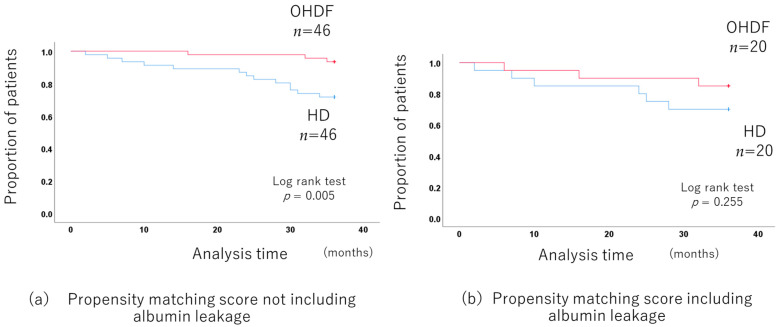
Comparison of the long-term prognosis between the HD and OHDF groups in patients more than 65 years old.

When comparing the OHDF and HD groups without including albumin leakage in propensity score matching, the 3-year prognosis was significantly better in the OHDF group. (*p* = 0.005) after adjusting for background factors by propensity score matching (46 pairs of HD and OHDF) (Figure 6a) (Supplementary Table S8).

There was no long-term prognosis benefit for the OHDF group when albumin leakage was included in the propensity score matching (*p* = 0.255 20 pairs of HD and OHDF) (Figure 6b).

The long-term prognosis was significantly worse for patients aged 65–79 years with an albumin leakage rate of less than 3.5 g (*p* = 0.008) and for patients aged over 80 years with an albumin leakage rate of less than 2.0 g (*p* = 0.008).

In the group of patients aged over 80 years, BMI (*p* = 0.036) and albumin leakage (*p* = 0.001) showed significant differences in terms of the long-term prognosis, while age (*p* = 0.774) and calorie intake (*p* = 0.102) did not.

## 4. Discussion

Comparing the HD and OHDF groups, there was better long-term prognosis in the OHDF group. However, the albumin leakage was significantly higher in the OHDF group. For similar levels of albumin leakage, there was no significant difference in prognosis between the HD and OHDF groups.

The results are similar when restricted to the group of patients more than 65 years old, who are more prone to nutritional disorders.

The prognosis improved with increased albumin leakage in both the HD and OHDF groups.

The improvement in the long-term prognosis with increased albumin leakage was more pronounced in the HD group compared to the OHDF group.

The effect was more pronounced in the group with low albumin leakage and tended to diminish slightly as the albumin leakage increased.

The long-term prognosis-improving effect of albumin leakage, therefore, levels off, and further albumin leakage worsens prognosis.

Hence, it is necessary to determine the optimal amount of albumin leakage.

The prognostic benefit of the high substitution volume in OHDF compared to HD has been reported [12,13].

The relationship between the substitution volume and the prognosis was not related to the long-term prognosis with substitution volumes of more than 60 L, as albumin leakage did not increase in proportion to the substitution volume in our data.

Therefore, the prognostic improvement may be due to increased albumin leakage and a more efficient removal of middle- to large-molecular-weight substances.

In Japan, it has been noted that 33 kda of α1-microgloblin is removed with albumin leakage, and our institution has confirmed the association between albumin leakage and α1-microgloblin removal (Figure S2). This result explains why the increase in albumin leakage is associated with the removal of medium molecular weight.

And the relationship between albumin leakage and α1-microgloblin removal did not differ between HD and OHDF.

The association between albumin leakage and prognosis involves the removal of uremic toxins.

In considering uremic toxins of prognostic relevance, it is necessary to understand that there are small-molecular-weight substances, middle-molecular-weight substances in the β2-microgloblin (β2-mg) region, and middle- to large-molecular-weight substances.

It is known that the removal of small-molecular-weight substances and β2-microgloblin (β2-mg) is related to long-term prognosis [12,13,14,15].

In small-molecular-weight uremic toxins, a Kt/V of 1.4 or higher is recommended, but raising it higher does not improve the prognosis [16].

β2-Mg can be removed in hemodialysis (HD) and online hemodiafiltration (OHDF) with the evolution of the dialysis membrane. Our facility uses dialysis membranes with a β2-Mg removal rate of more than 70%, which is sufficient for removal efficiency in both HD and OHDF.

It should also be noted that there is no association between β2-Mg removal and albumin leakage. HDF can remove middle-to-large-molecular-weight substances more efficiently than HD [17].

It was recently shown that both HD and OHDF can remove β2-MG efficiently, which means that β2-MG cannot be used as a biomarker to accurately assess the removal efficiency of online HDF.

Middle molecular weight substances can be subdivided into three categories (small–middle-weight molecules of 0.5–15 kDa, medium–middle weight molecules of 15–25 kDa, and large–medium-weight molecules of 25–58 kDa) [18]. Middle- to large-molecular-weight substances are important in uremic toxins associated with albumin leakage. For example, IL-1 and IL-6 [19], parathyroid hormone, prolactin, and osteocalcin are associated with chronic inflammation and the prognosis of dialysis patients.

Recently, κ free light chains (22,000 Da) and λ free light chains (42,000 Da) have been identified as middle-molecular-weight uremic toxins [20].

The removal of middle- to large-molecular-weight substances may also be involved in the reduction in inflammatory cytokines in hemodialysis patients [20,21].

Albumin leakage is not only an indicator of the removal of middle-to-large-molecular-weight substances, but it is also related to the nutritional status, and the assessment of the nutritional status is essential for increasing albumin leakage.

Albumin production is related to energy intake and blood amino acid levels.

Patients with a good nutritional status can tolerate dialysis that leaks albumin and can benefit from the albumin leakage.

The serum albumin level is determined by the rate of synthesis in the liver, its distribution among body compartments, its catabolism, and external losses, such as during dialysis [22].

The serum albumin in hemodialysis patients has a prolonged half-life and appears to be degraded and denatured.

Hemodialysis patients have an increased albumin synthesis capacity compared to healthy subjects and need to try to maintain serum albumin levels [23].

The albumin synthesis capacity is further increased in dialysis patients when hypoalbuminemia is present [24].

Increased albumin leakage stimulates albumin synthesis and increases the mercaptoalbumin ratio [25], which also improves the albumin quality.

As albumin is synthesized in the liver, conditions such as cirrhosis also need attention.

Therefore, it is necessary to determine the albumin leakage that is acceptable for each individual patient.

There are risks and benefits of albumin leakage, and it is important to find the best balance between them.

### 4.1. Nutritional Status and Dietary Intake Are Related

The guidelines of the Japanese Society for Dialysis Therapy recommend an energy intake of 30–35 Kcal/kg and a protein intake of 0.9–1.2 g/kg.

However, elderly dialysis patients over 65 years of age are more likely to have nutritional problems as dietary intake declines.

Therefore, elderly dialysis patients need to increase their dietary intake to improve their nutritional status.

There was a significant positive correlation between dietary intake and albumin leakage (r = 0.21, *p* = 0.020).

Serum albumin is no longer a prognostic indicator because of the aggressive albumin leakage in well-nourished patient groups.

Hypoalbuminemia due to malnutrition has a poor prognosis, and albumin leakage should be minimized.

In contrast, in well-nourished patients, it may be useful to increase the albumin leakage to the maximum tolerated level.

We believe that low serum albumin levels associated with albumin leakage are not necessarily a risk factor [9].

Albumin leakage may also be involved in improved dietary intake due to leptin removal [26].

Therefore, in the long term, improved nutritional status can be expected.

However, in the short term, there is a risk of a fall in the serum albumin levels and the deterioration in the nutritional status.

Serum albumin levels should be maintained at least at 3.0–3.2 g/dL as a result of albumin leakage.

For this value, we refer to the fact that the average blood albumin level of peritoneal dialysis patients in our hospital remains around 3.1 g/dL.

Increasing dietary intake in patients over 80 years of age is a difficult problem, and in this study, the ability to tolerate albumin leakage of more than 2 g was a survival boundary.

As each patient has a different pathology, it is important to understand the patient’s condition, taking into account factors such as age, rather than just looking at blood data.

### 4.2. Albumin Leakage in Japanese-Style OHDF

Albumin leakage in pre-OHDF is safer than in post-HDF under the condition of high albumin leakage.

In Japan, where pre-OHDF is the main method, a wide range of membranes are used, from high albumin leakage to low albumin leakage.

In Europe, on the other hand, post-OHDF is the standard, and the membrane types are limited, with MCO and HCO membranes leaking albumin. MCO and HCO membranes have been reported to be effective in removing the middle- to large-molecular-weight uremic toxins.

However, albumin leakage in MCO membranes is limited to around 3 g [27], and HCO membranes are at risk of albumin leakage of more than 30 g.

Therefore, albumin leakage is limited in Europe. Against this background, some reports in Europe suggest that albumin leakage of around 4 g is acceptable [26].

However, Japanese-style pre-OHDF can be safely performed even when the target albumin leakage is more than 4 g.

This study showed that there are patients with albumin leakage of more than 6.5 g who have an improved prognosis, indicating that aggressive albumin leakage may be useful in some patients. It is important to determine the tolerable amount of albumin leakage based on the patient’s general condition, including nutritional status, without the aim of increasing the amount of albumin leakage.

### 4.3. Limitations

This study was a single-center retrospective study with a small number of cases.

However, it is also important to note that this was a single-center study, with standardized therapy selection and dialysis conditions and no facility-to-center bias. Therefore, even though the number of cases is small, it is still a meaningful study.

The presence of selection bias between HD and OHDF was considered when comparing the patient backgrounds.

Patients with hemodialysis vascular access failure or circulatory instability may be selected for HD due to the quantity of blood flow (QB) associations. Selection bias may exist in other factors as well, and this is also the case in the selection of pre-OHDF and post-OHDF. However, the current study included patients who were able to attend as outpatients and did not include patients with extremely poor nutritional status.

This was a one-point study, and subsequent changes in dialysis conditions could not be taken into account. The majority of patients had no change in dialysis method (HD or OHDF).

It is also possible that some items were propensity-score-matched according to previous reports but were not adjusted.

Albumin leakage is slightly different in every dialysis, even under the same dialysis conditions.

This is seen not only with albumin leaks but also with other uremic toxins.

The problem is that albumin leakage can only be assessed by measurement, whereas with small-molecular-weight substances, they can be assessed by Kt/v.

Of course, it also depends on factors such as the dialysis conditions, amount of replacement fluid, dialysate flow rate, dialysis membrane, size of the membrane, etc., and therefore, needs to be assessed for each condition.

The measurement method requires measuring the drainage from the dialysate, which is costly and consuming.

It is, therefore, not possible to measure it on every dialysis, and assessment with an estimated albumin leak is realistic. It is also unclear whether albumin leakage is the best marker for the removal of middle- to large-molecular-weight substances.

In addition, there are limitations in comparing patients with different albumin leakage tolerances.

The removal of uremic toxins within the tolerable range of albumin leakage is useful in biological defense, but its effectiveness also varies between individuals. There is also no way of knowing which uremic toxins are most effectively removed as middle- to large-molecular-weight substances in albumin leakage.

There is also no precise measurement to assess the balance between targets for the removal of dietary uremic toxins, uremic toxins causing chronic inflammation, inflammatory cytokines associated with chronic inflammation, etc., and the tolerated removal dose, which depends on the albumin-producing capacity. This calls for the setting of effective dialysis conditions within the tolerances of individual patients.

## 5. Conclusions

The results suggest that survival is improved more with high albumin leakage than with low albumin leakage in both OHDF and HD patients.

In well-nourished patients, albumin neogenesis in the liver is good, but the accumulation of uremic toxins is also high; thus, albumin leakage should be increased and removed as much as possible. This leads to the elimination of uremic toxins, which improves long-term prognosis.

The aggressive removal of middle- to large-molecular-weight substances at a time when high albumin leakage can be tolerated is important.

The main purpose of albumin leakage is to promote turnover and to remove middle- to large-molecular-weight uremic toxins.

The present results show that albumin leakage is one of the useful indicators of middle- to large-molecular-weight substance removal. The balance between serum albumin levels and albumin leakage needs further investigation.

## Figures and Tables

**Table 1:** Background factors before and after propensity score matching in the HD and OHDF groups.

	Before Matching			After Matching		
	HD (*n* = 159)	OHDF (*n* = 301)	*p* Value	HD (*n* = 137)	OHDF (*n* = 137)	*p* Value
Age	72.1 ± 11.8	64.2 ± 11.8	<0.001	70.7 ± 11.3	68.9 ± 11.3	0.208
Dialysis vintage (years)	8.8 ± 8.9	11.4 ± 9.8	0.006	9.2 ± 8.9	9.6 ± 8.1	0.656
Diabetes mellitus	50/159	95/301	0.842	79/137	75/137	0.626
BMI (kg/m^2^)	21.8 ± 3.7	22.8 ± 3.9	0.005	21.9 ± 3.8	22.5 ± 3.9	0.168
Blood pressure (mmHg)	143 ± 25	143 ± 25	0.864	143 ± 25	141 ± 25	0.641
Kt/V	1.61 ± 0.33	1.57 ± 0.30	0.174	1.61 ± 0.34	1.57 ± 0.29	0.408
nPCR (g/kg/day)	0.83 ± 0.20	0.87 ± 0.15	0.035	0.84 ± 0.20	0.86 ± 0.16	0.513
Hb (g/dL)	11.0 ± 1.3	11.4 ± 1.1	0.004	11.1 ± 1.3	11.1 ± 1.1	0.764
Alb (g/dL)	3.27 ± 0.39	3.37 ± 0.27	0.006	3.31 ± 0.36	3.33 ± 0.26	0.513
Ca (mg/dL)	8.7 ± 0.8	8.8 ± 0.7	0.161	9.5 ± 0.7	9.4 ± 0.7	0.444
P (mg/dL)	5.2 ± 1.6	5.5 ± 1.4	0.031	5.3 ± 1.5	5.2 ± 1.2	0.704
HS-CRP (mg/dL)	2.28 ± 2.77	2.42 ± 2.86	0.604	2.36 ± 2.84	2.67 ± 3.04	0.393
β2-MG (mg/L)	27.5 ± 7.1	27.6 ± 6.2	0.870	27.6 ± 7.1	27.0 ± 6.5	0.433
Albumin leakage (g)	2.14 ± 1.16	4.92 ± 2.79	<0.001	2.23 ± 1.13	4.38 ± 2.62	<0.001

## Data Availability

All data generated or analyzed during this study are included in this published article and its Supplementary Materials.

## Supplementary Materials

The following supporting information can be downloaded at https://www.mdpi.com/article/10.3390/jcm13071865/s1: Figure S1: Substitution volume and long-term prognosis in pre-OHDF; Figure S2: Relationship between albumin leakage and α1-microgloblin removal; Table S1a,b: Dialysis condition and albumin leakage in HD and OHDF groups; Table S2: Background factors before and after propensity score matching in HD (albumin leakage of >3) and OHDF groups; Table S3: After adjusting for background factors of HD group by albumin leakage; Table S4: After adjusting for background factors of OHDF group by albumin leakage; Table S5: After adjusting for background factors of pre-OHDF group by albumin leakage; Table S6: Patient background by substitution volume in pre-OHDF group; Table S7: Background factors of post-OHDF group by albumin leakage; Table S8: Background factors before and after propensity score matching in HD and OHDF groups where dietary intake could be evaluated.
