# Supplementary material for: Impact of Albumin Leakage on the Mortality of Patients Receiving Hemodialysis or Online Hemodiafiltration
Source: J Clin Med. 2024 Mar 24;13(7):1865. doi: 10.3390/jcm13071865 (PMC11012308; doi:10.3390/jcm13071865)
Supplement: Supplementary file 1 [file jcm-13-01865-s001.zip › jcm-2905065-supplementary.pdf]

Hemodiafilters		Mean albumin leakage(g/session)						
		Pre-OHDF			Post-OHDF			
Material	Product name	60L	72L	84L	8L	10L	12L	16L
CTA: cellulose triacetate	FIX 250Eeco	0.8	0.8	0.9	1.1	1.2	1.1	1.4
	FIX 250Seco	3.8	3.5	3.4	4.2	4.5	5.8	5.0
PEPA:polyester polymer alloy	GDF-21	11.1	14.1	16.9	8.6	10.5	12.6	14.4
PES: polyethersulfone	MFX-25Ueco	5.2	6.9	7.0	5.9	6.9	7.2	9.9
	MFX-30Ueco	4.4	4.5	5.2	8.3	8.7	12.4	16.3
PS: polysulfone	ABH-22PA	2.5	2.8	4.6	2.1	2.8	4.6	6.2
	ABH-26PA	2.3	2.7	3.1	3.1	3.6	5.3	9.8
	NVF-21M	0.9	0.9	1.2	0.8	0.9	1.0	1.8
	NVF-26M	0.6	0.6	0.9	0.8	0.8	1.0	1.8
	NVF-26P	1.9	2.2	2.9	2.8	3.5	4.1	5.8

Table S1a
Dialysis condition and Estimated albumin leakage in OHDF group.

Dialyzers		
Material	Product name	Mean albumin leakage(g/session)
CTA: cellulose triacetate	FB-150Uβ eco	0.61
PEPA: polyester polymer alloy	FDZ-21	2.12
PES: polyethersulfone	PES-21Dα eco	4.08
	PES-25Dα eco	4.53
PMMA: polymethyl-methacrylate	NF-2.1H	3.22
PS: polysulfone	APS-21EA	2.27
	APS-25EA	1.98
	NV-21X	1.92
	PN-220S	1.73

Table S1b Dialysis condition and Estimated albumin leakage in HD group.

	Before matching			After matching		
	HD(n=37)	OHDF(n=301)	P-value	HD (n=37)	OHDF (n=37)	P-value
Age	63.9±10.2	64.2±11.8	0.854	63.9±10.2	67.3±12.7	0.224
Dialysis vintage (years)	9.4±8.6	11.4±9.8	0.263	9.4±8.6	11.4±9.1	0.336
diabetes mellitus	14/37	95/301	0.392	14/37	10/37	0.321
BMI (kg/m²)	23.2±4.1	22.8±3.9	0.578	23.2±4.1	22.5±3.4	0.417
Blood pressure(mmHg)	147±23	143±25	0.372	147±23	141±25	0.323
Kt/V	1.57±0.33	1.57±0.30	0.889	1.57±0.33	1.56±0.36	0.989
nPCR (g/kg/day)	0.88±0.18	0.87±0.15	0.686	0.88±0.18	0.85±0.14	0.439
Hb (g/dL)	11.0±1.2	11.4±1.1	0.076	11.0±1.2	11.5±1.2	0.099
Alb (g/dL)	3.42±0.33	3.37±0.27	0.274	3.42±0.33	3.38±0.30	0.627
Ca (mg/dL)	8.7±0.8	8.8±0.7	0.509	8.7±0.8	8.7±0.7	0.983
P (mg/dL)	5.6±1.4	5.5±1.4	0.722	5.6±1.4	5.1±1.3	0.168
HS-CRP(mg/dL)	2.49±2.80	2.42±2.86	0.911	2.49±2.80	2.43±2.91	0.930
β2-MG(mg/L)	28.0±7.3	27.6±6.2	0.694	28.0±7.3	25.9±4.5	0.133
Albumin leak (g)	3.92±0.46	4.92±2.79	<0.001	3.92±0.46	4.01±1.55	0.728

Table S2.
Background factors before and after propensity scores matching in HD(Albumin leak >3) and OHDF groups.

Albumin leakage	<1g (n=13)	1-3g (n=13)	<3g (n=13)	P-value
Age	70.0±9.8	68.5±12.2	69.6±9.6	0.929
Dialysis vintage (years)	10.1±12.4	7.0±7.2	9.9±6.4	0.629
diabetes mellitus	4/13	5/13	4/13	0.891
BMI (kg/m²)	19.7±3.2	21.9±4.1	23.1±3.3	0.060
Blood pressure(mmHg)	130±39	133±19	139±22	0.735
Kt/V	1.70±0.39	1.68±0.22	1.57±0.43	0.656
nPCR (g/kg/day)	0.84±0.20	0.81±0.19	0.94±0.20	0.242
Hb (g/dL)	10.6±0.9	11.4±0.9	11.4±0.8	0.055
Alb (g/dL)	3.37±0.30	3.18±0.50	3.29±0.27	0.428
Ca (mg/dL)	8.7±0.7	8.7±1.0	8.4±0.4	0.542
P (mg/dL)	5.6±1.1	4.8±1.7	5.4±1.1	0.290
HS-CRP(mg/dL)	1.90±2.17	2.10±3.22	3.33±3.0	0.437
β2-MG(mg/L)	25.2±9.3	30.4±7.1	28.9±7.9	0.268
Albumin leak (g)	0.52±0.21	1.88±0.15	3.82±0.52	<0.001

Table S3.
After Adjusted background factors for HD group by albumin leakage.

Albumin leakage	<3.5g (n=56)	3.5-6.5g (n=56)	<6.5g (n=56)	P-value
Age	63.1±10.5	62.7±12.6	62.8±9.3	0.979
Dialysis vintage (years)	11.6±11.0	11.8±11.0	13.1±9.7	0.709
diabetes mellitus	22/56	13/56	14/56	0.122
BMI (kg/m²)	23.1±4.7	23.3±3.1	22.8±3.8	0.787
Blood pressure(mmHg)	141±22	140±23	148±23	0.136
Kt/V	1.54±0.30	1.54±0.31	1.58±0.28	0.735
nPCR (g/kg/day)	0.85±0.15	0.86±0.16	0.88±0.13	0.542
Hb (g/dL)	11.3±1.0	11.1±0.9	11.0±1.0	0.371
Alb (g/dL)	3.37±0.30	3.36±0.27	3.38±0.30	0.909
Ca (mg/dL)	8.7±0.7	8.8±0.7	8.9±0.8	0.498
P (mg/dL)	5.3±1.2	5.5±1.6	5.7±1.4	0.403
HS-CRP(mg/dL)	2.31±2.35	2.35±2.74	2.18±2.67	0.939
β2-MG(mg/L)	27.4±6.0	27.2±6.5	28.0±5.5	0.758
Albumin leak (g)	2.34±1.03	4.53±0.63	7.65±1.71	<0.001

Table S4.
After Adjusted background factors for OHDF group by albumin leakage.

Albumin leakage	<3.5g (n=35)	3.5-6.5g (n=35)	<6.5g (n=35)	P-value
Age	63.2±11.9	60.9±13.9	63.5±7.9	0.596
Dialysis vintage (years)	11.5±10.9	10.7±9.2	13.3±9.1	0.517
diabetes mellitus	23/35	21/35	18/35	0.396
BMI (kg/m²)	22.5±4.3	24.1±4.0	23.1±3.3	0.243
Blood pressure(mmHg)	142±23	147±23	149±22	0.435
Kt/V	1.59±0.28	1.45±0.31	1.53±0.27	0.108
nPCR (g/kg/day)	0.85±0.16	0.84±0.11	0.90±0.13	0.156
Hb (g/dL)	11.4±1.2	11.4±0.9	11.0±1.1	0.167
Alb (g/dL)	3.40±0.36	3.41±0.31	3.39±0.28	0.981
Ca (mg/dL)	8.8±0.6	8.9±0.8	9.0±0.7	0.569
P (mg/dL)	5.2±1.2	5.7±1.6	5.6±1.6	0.378
HS-CRP(mg/dL)	2.32±2.40	2.30±2.77	2.34±2.67	0.998
β2-MG(mg/L)	27.8±6.3	27.2±6.3	28.7±5.8	0.581
Albumin leak (g)	2.04±1.03	4.53±0.63	7.65±1.71	<0.001

Table S5. After Adjusted background factors for Pre-OHDF group by albumin leakage.

	60 L(n = 83)	72 L(n = 11)	84 L(n = 117)	P-value
Age	64.5±12.5	64.8±14.0	66.0±11.5	0.629
Dialysis vintage (years)	10.6±9.8	14.2±7.7	10.2±8.2	0.368
diabetes mellitus	50/83	5/11	75/117	0.452
BMI (kg/m²)	22.9±4.0	24.1±4.5	23.3±4.0	0.628
Blood pressure(mmHg)	140±25	149±26	146±24	0.196
Kt/V	1.52±0.30	1.56±0.32	1.52±0.29	0.933
nPCR (g/kg/day)	0.87±0.15	0.86±0.17	0.87±0.15	0.906
Hb (g/dL)	11.4±1.2	11.0±1.0	11.4±1.1	0.550
Alb (g/dL)	3.40±0.31	3.42±0.33	3.36±0.24	0.683
Ca (mg/dL)	9.4±0.7	9.8±0.6	9.3±0.6	0.091
P (mg/dL)	5.5±1.5	5.9±2.1	5.5±1.2	0.583
HS-CRP(mg/dL)	2.21±2.76	2.99±3.73	2.41±2.77	0.664
β2-MG(mg/L)	28.5±5.9	29.9±8.8	27.0±6.2	0.103
Albumin leak (g)	3.47±2.48	5.85±4.34	4.44±1.88	0.001

Table S6. Patient background by substitution volume in pre-OHDF group.

Albumin leakage	<5g (n=28)	5-7g (n=25)	<7g (n=37)	P-value
Age	64.8±9.3	61.8±12.2	58.1±11.6	0.101
Dialysis vintage (years)	15.6±14.4	11.4±9.6	12.9±10.7	0.369
diabetes mellitus	9/28	17/25	11/37	0.717
BMI (kg/m²)	21.3±3.4	21.6±2.7	23.4±4.5	0.058
Blood pressure(mmHg)	139±28	146±25	143±23	0.568
Kt/V	1.74±0.26	1.63±0.26	1.55±0.27	0.034
nPCR (g/kg/day)	0.88±0.15	0.88±0.16	0.87±0.15	0.924
Hb (g/dL)	11.4±1.1	11.5±0.9	11.2±0.9	0.615
Alb (g/dL)	3.35±0.25	3.35±0.27	3.32±0.26	0.903
Ca (mg/dL)	8.7±0.7	9.1±0.6	8.8±0.8	0.087
P (mg/dL)	5.7±1.4	5.2±1.1	5.8±1.4	0.128
HS-CRP(mg/dL)	1.52±2.35	2.88±2.84	3.19±3.63	0.029
β2-MG(mg/L)	26.7±6.6	27.1±6.9	28.1±5.2	0.685
Albumin leak (g)	4.05±0.70	6.19±0.47	9.96±2.80	<0.001

Table S7. Background factors for Post-OHDF group by albumin leakage.

	Before matching			After matching		
	HD(n=46)	OHDF(n=93)	P-value	HD (n=46)	OHDF (n=46)	P-value
Age	76.7±7.9	72.1±6.2	0.001	76.7±7.9	74.5±7.1	0.161
Dialysis vintage (years)	8.9±9.3	11.0±10.2	0.230	8.9±9.3	8.6±9.0	0.874
diabetes mellitus	8/46	28/93	0.107	8/46	11/46	0.440
BMI (kg/m²)	21.3±3.0	22.1±3.4	0.203	21.3±3.0	21.6±3.6	0.644
Blood pressure(mmHg)	141±24	141±25	0.992	141±24	141±27	0.951
Kt/V	1.57±0.33	1.57±0.30	0.889	1.68±0.33	1.64±0.29	0.542
nPCR (g/kg/day)	0.88±0.18	0.87±0.15	0.686	0.84±0.23	0.89±0.16	0.222
Hb (g/dL)	11.0±1.2	11.4±1.1	0.076	11.1±1.1	11.1±1.0	0.907
Alb (g/dL)	3.42±0.33	3.37±0.27	0.274	3.24±0.25	3.28±0.21	0.418
Potassium(mEq/L)	4.6±0.7	4.9±0.6	0.034	4.6±0.7	4.9±0.7	0.070
Ca (mg/dL)	8.7±0.8	8.8±0.7	0.509	8.7±0.6	8.7±0.6	0.852
P (mg/dL)	5.6±1.4	5.5±1.4	0.722	5.0±1.4	5.2±1.2	0.331
HS-CRP(mg/dL)	2.49±2.80	2.42±2.86	0.911	2.36±3.06	2.63±3.05	0.674
β2-MG(mg/L)	28.0±7.3	27.6±6.2	0.694	27.8±7.6	27.6±5.5	0.883
Albumin leak (g)	3.92±0.46	4.92±2.79	<0.001	1.83±0.79	4.12±2.29	<0.001
Calories(Kcal)	1573±382	1659±360	0.199	1573±383	1647±399	0.368
Protein(g)	58.0±16.8	60.8±14.1	0.315	58.0±16.8	61.2±14.2	0.327
Potassium(mg)	1653±532	1791±481	0.129	1653±532	1828±512	0.111
Phosphorus(mg)	814±252	843±190	0.493	814±252	856±198	0.374
Salt(g)	6.6±2.0	6.9±2.0	0.392	6.6±2.0	6.9±2.2	0.540

Table S8.
Background factors before and after propensity score matching in HD and OHDF groups where dietary intake could be evaluated.

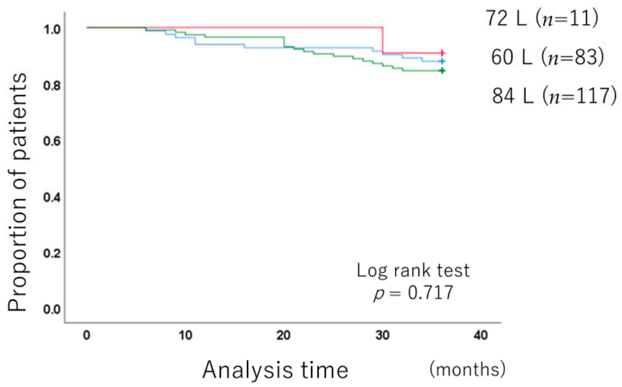

Figure S1. Substitution volume and long-term prognosis in Pre-OHDF.

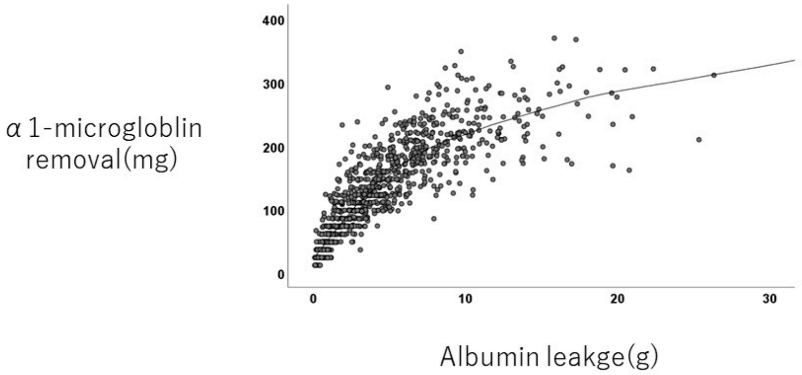

Figure S2. Relationship between albumin leakage and α1-microgloblin removal.
